# Complicated Role of Post-translational Modification and Protease-Cleaved Fragments of Tau in Alzheimer’s Disease and Other Tauopathies

**DOI:** 10.1007/s12035-023-03867-x

**Published:** 2023-12-20

**Authors:** Jie Yang, Naiting Shen, Jianying Shen, Ying Yang, Hong-Lian Li

**Affiliations:** 1 Tongji Hospital, Tongji Medical College, Huazhong University of Science and Technology, Wuhan, 430030 China; 2 Department of Histology and Embryology, School of Basic Medicine, Key Laboratory of Education Ministry, Hubei Province of China for Neurological Disorders, Tongji Medical College, Huazhong University of Science and Technology, Wuhan, 430030 China; 3 Department of Pathophysiology, School of Basic Medicine, Key Laboratory of Education Ministry, Hubei Province of China for Neurological Disorders, Tongji Medical College, Huazhong University of Science and Technology, Wuhan, 430030 China

**Keywords:** Post-translational modification of tau, Tau fragment, Alzheimer’s disease

## Abstract

Tau, a microtubule-associated protein predominantly localized in neuronal axons, plays a crucial role in promoting microtubule assembly, stabilizing their structure, and participating in axonal transport. Perturbations in tau’s structure and function are implicated in the pathogenesis of neurodegenerative diseases collectively known as tauopathies, the most common disorder of which is Alzheimer’s disease (AD). In tauopathies, it has been found that tau has a variety of post-translational modification (PTM) abnormalities and/or tau is cleaved into a variety of fragments by some specific proteolytic enzymes; however, the precise contributions of these abnormal modifications and fragments to disease onset and progression remain incompletely understood. Herein, we provide an overview about the involvement of distinctive abnormal tau PTMs and different tau fragments in the pathogenesis of AD and other tauopathies and discuss the involvement of proteolytic enzymes such as caspases, calpains, and asparagine endopeptidase in mediating tau cleavage while also addressing the intercellular transmission role played by tau. We anticipate that further exploration into PTMs and fragmented forms of tau will yield valuable insights for diagnostic approaches and therapeutic interventions targeting AD and other related disorders.

## Introduction

Alzheimer’s disease (AD), a progressive neurodegenerative brain disease featured by cognitive impairment and memory loss, has become the most common reason for dementia among the elderly [1]. AD has two main features: the formation of β-amyloid plaques and hyperphosphorylated tau-caused neurofibrillary tangles (NFTs) [2, 3]. Tau is a microtubule-associated protein (MAP) present in neurons that improves microtubule assembly, stabilizes microtubules, and participates in axonal transport [4–7]. Studies found that tau, as a synaptic protein, had synaptic functions to promote dendrite elongation, spine formation, and synaptic plasticity [8, 9]. The abnormal misfolding of tau protein forms β-folded fibrils that aggregate in the central nervous system (CNS) neurons to form tau aggregates, leading to neurodegenerative diseases collectively termed as tauopathies [10]. Disorders included in this category are AD, progressive supranuclear palsy (PSP), corticobasal degeneration (CBD), argyrophilic grain disease, aging-associated tau astrocytosis, and primary age-related tauopathies [11, 12].

Under pathological conditions, Tau aggregation begins with tau detaching from microtubules, leading to microtubule disassembly. The detached tau protein aggregates to generate oligomers, which in turn form paired helical filaments (PHFs) with a double-helix bond structure, and finally form NFTs [13]. Tau affinity for tubulin is modulated by post-translational modifications (PTMs) of tau [13, 14], which include phosphorylation, glycosylation, O-linked *N*-acetylglucosamine (O-GlcNAc) glycosylation, acetylation, methylation, oxidation, nitrification, ubiquitination, and SUMOylation. Tau phosphorylation has been the most studied so far.

In addition to PTMs mentioned above, tau truncation is another important modification mediated by specific proteases that promote neurodegenerative variations via neurotoxic tau fragments that can aggregate and/or propagate across cells. To date, a number of proteolytic fragments of tau have already been identified in cerebrospinal fluid (CSF) and plasma from patients with neurodegenerative diseases, supporting their application as biomarkers of disease progression [15]. At present, there are corresponding therapeutic strategies to inhibit the toxicity of truncated tau, such as repressing protease activity, selectively weakening protease-substrate interaction, and preventing truncated tau from acting [16]. However, the protein breakdown process and pathological changes caused by the transformation of each tau isoform to distinct tau fragments remain to be further studied.

Therefore, this review aims to comprehensively understand PTMs of tau and has a special focus on protease-mediated tau truncation, including their formation process, distribution location, possible pathogenic mechanism, and pathological impacts. Furthermore, we also discuss tau fragments in intercellular secretion and transmission. It is desired to find the action points of inhibiting tau lesions and provide crucial targets for the diagnosis and treatment of tauopathies including AD.

## Molecular Structure and Functions of Tau

**Fig. 1:**
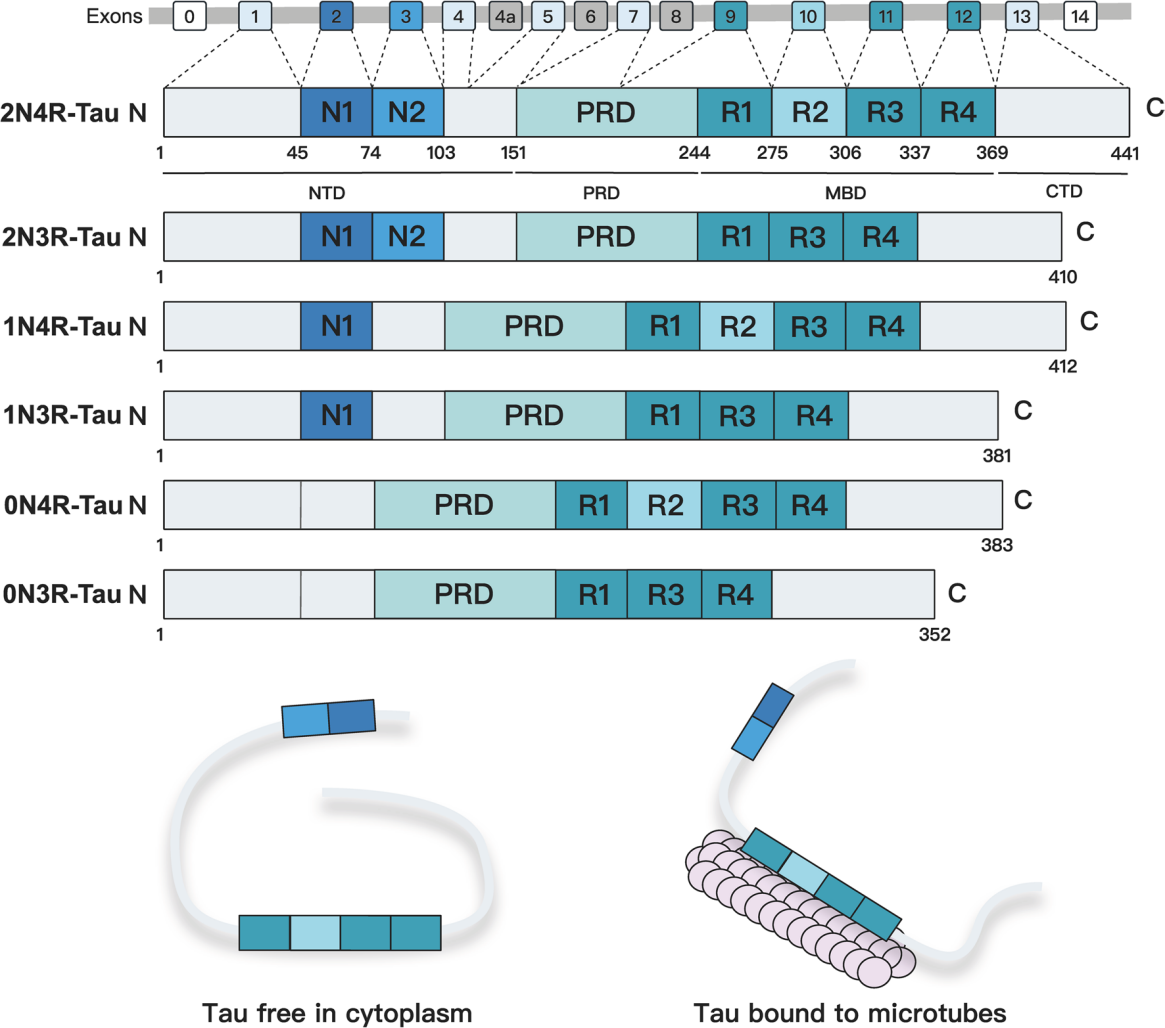
Organization of tau gene and isoforms of tau protein

Human tau is a MAP encoded by the MAPT gene, which is located on chromosome 17 and consists of 16 exons [17]. Tau is overall hydrophilic with its relatively low proportion of hydrophobic amino acids and thus has a high degree of thermal stability and solubility [18]. Tau can be subdivided into four functional domains: an N-terminal projection domain (amino acids 1–150), a proline-rich domain (PRD) encompassing residue 151–243, a microtubule-binding domain (MBD) encompassing residue 244–369, and a C-terminal region (amino acids 370–443), which are characterized by their diverse biochemical properties [19] (Fig. 1). Microtubule binding repeat sequences in MBD (termed R) and N-terminal exons (termed N) together determine the type and naming of six different major tau isoforms, and the expression of these isoforms is developmentally modulated [20]. In adults, all six main isoforms of tau are formed by alternative splicing around MBD and the N-terminal region in the CNS, while only 0N3R isoform is present in fetal brain [20]. The second and third MBD repeats tend to exhibit a well-ordered β-sheet structure [21]. Exon 10 encodes the R2 fragment, and thus, tau with exon 10 in MBD is collectively referred to as 4R tau, and vice versa as 3R tau. In the brains of adult humans, the proportion of 3R tau and 4R tau is equivalent, while there is only 4R tau in adult mice [17, 22]. Collectively, tau expresses in six main distinct isoforms in the human CNS via alternative splicing processes, and alternative splicing around exon 6 produces six additional isoforms [23]. For instance, a set of additional tau isoforms 6D and 6P generated by the alternative splicing of exon 6 may ameliorate the polymerization of full length tau (htau40) and thus are promising endogenous inhibitors of filamentous tau formation [24].

Tau expression exists throughout the whole brain development, and six isoforms of tau eventually exist in human embryonic stem cell-derived neurons [25]; hence, severity of the increase in tau mRNA levels is synchronized with the degree of maturation of cells [26]. Specifically, tau expression levels are high in SYN + /TBR1 + mature neurons and low in intermediate precursor cells in deep white matter and radial glia in the subventricular zone, with minimal expression in the germinal matrix and subventricular zone [26]. Tau shares a similar expression pattern with induced pluripotent stem cell–derived cortical organisms, and both increase with neuronal maturation, being a promising target for the therapy of neurodevelopmental disorders [26, 27]. Under pathological conditions, tau may accumulate in the perinuclear region of neurons or be expressed in certain neuronal populations. For instance, all the six isoforms of tau exist in dystrophic olfactory epithelial neurites of AD patients [28].

Biophysical studies have shown that tau is a soluble unfolded protein with little secondary structure but highly flexible conformation under physiological conditions [29]. However, tau can perform its tertiary structure when tau is separated from microtubules: “paperclip” conformational folding via intramolecular interactions between charged regions, with the C-terminus folded on MBD and the N-terminus folded on the C-terminus [30–32]. Recent studies have revealed that phosphatase activation domain (PAD), the N-terminus region spanning amino acid 2–18, is tucked away beneath the native protein in a paperclip conformation. Aberrant modification of tau is likely to alter its conformation through abnormal exposure of PAD, thereby thwarting normal biological functions of tau [33] and exerting prominent effects on inhibiting tau aggregation and rapid axonal transport [34]. Proline-directed tau phosphorylation also tends to impair tau conformation, loosing or tightening its paperclip conformation [35]. The unfolding of the paperclip conformation may occur before tau oligomers and NFTs form, giving a hint that the unfolded paperclip is a precursor from NFTs [36].

As a multifunctional protein, tau can not only regulate microtubule dynamics and influence cytoskeletal components but also modulate signaling pathways through serving as a protein scaffold for signaling cascades. Numerous studies have found that tau has a novel and important role in multiple physiological functions.

Tau protein, as MAP, combines with microtubules to maintain neuronal health. The prevailing view is that the crucial physiological function of tau in neurons is to stabilize microtubules in axons. A new opinion suggested that tau do not stabilize axon microtubules but promote the assembly of labile domains while restricting its binding to microtubule stabilizers, resulting in long labile regions of axon microtubules [37]. The binding of tau to microtubules is interfered by PTMs, inducing the pathological aggregation of tau. Recently, one pseudo-repeat sequence R’ of MBD of tau were targeted. R’ outperforms the other four repeat sequences when combining to microtubules. Since R’ contains the most charged residues, charge-charge interactions could promote the combination of tau and microtubules [38]. Additionally, MAP has a pivotal role in the modulation of neuronal microtubule cytoskeleton and dynamics, among which tau is the most enriched. Tau bundles and cross-links actin filaments.

Tau is also located in the nucleus of neurons. It is mainly expressed in the nucleolus and pericentromeric heterochromatin (PCH), and nuclear chromatin proteins like tau may modulate chromosome stability and transcription. Tau could interact with nuclear lamina; hence, the nuclear transport in neurons would be influenced under pathogenic conditions [39]. In the nucleolus, the co-localization was found between tau and the related nucleolus proteins, for example, epigenetic factor BAZ2Aor upstream binding transcription factor [40], which suggests that tau may be involved in rDNA transcription and ribosome synthesis. Furthermore, it has been found that tau’s binding to the promotor region of rDNA locus could act as a potential role in regulating transcriptional processes [41]. By utilizing a tau gene knockout mouse model to alter tau expression, its involvement in transcriptional regulation could possibly confirmed. Nuclear tau may act as a protective role during stressful events, since PCH heat shock–induced DNA repair was damaged in tau KO mice [42].

The significance of tau in maintaining the normal structure and function of neurons has been further elucidated through extensive research, highlighting its multifaceted roles and indispensability.

## Role of Post-translational Modification of Tau in Tauopathies

The affinity of tau to tubulin is regulated by various PTMs, encompassing phosphorylation, glycosylation, acetylation, methylation, oxidation, nitration, ubiquitination, and SUMOylation. Among these PTMs studied extensively thus far is tau phosphorylation. Acetylation and some other PTMs also impair the functions of tau and promote tau monomer aggregation [43]. The study of the pathological mechanisms of major PTMs is helpful for the targeted treatment of tauopathies.

### Phosphorylation

Numerous amino acids of tau are potential phosphorylation sites. Tau phosphorylation is regulated by the activities of protein kinase and phosphatase. A large number of studies have shown that the activity of multiple protein kinases in the brain region of AD patients increases, while the activity of phosphatase decreases [44, 45]. A certain degree of phosphorylation of tau in its normal state does not interfere with the affinity with microtubules, but hyperphosphorylation can separate tau from microtubules in cell [46, 47] and destabilizes microtubules by making itself more likely to aggregate into insoluble inclusions [48]. Eventually, filamentous, insoluble tau aggregates and aggregated PHFs are formed, namely NFTs [49].

In recent years, many researches devoted to exploring which sites of tau phosphorylation can reduce its affinity for microtubules. Some of the known phosphorylation sites that alter microtubule stability are S258, S262, S324, and S356 [50–52]. A recent tau structure analysis done by Brotzakis et al. found that S262, S324, and S356 all had an effect on tau-microtubule complex stability, and among these sites, S262 had a relatively more significant effect [52]. Protein kinase R (PKR)-mediated phosphorylation of Thr181, Ser199/202, Thr231, Ser262, Ser396, Ser404, and Ser409 could remove tau from intracellular microtubules [53]. Tyrosine sites, which are less numerous than the other two amino acids, can also be phosphorylated but may also play a stronger role in the affinity between tau and microtubules. Phosphorylation of multiple N-terminal tyrosine residues, or specific phosphorylation at the residue of tyr-310 only, locally reduces the β-sheet tendency structural domain of tau in PHF6, thereby eliminating tau aggregation and inhibiting its properties of microtubule binding [54, 55]. Phosphorylation of Ser396-404, present in 50% of the total structure of early phosphorylated tau aggregates in brain sections from AD and Down syndrome patients [56], is also thought to be more important for tau events since being found to reduce the solubility of full-length tau and promote aggregation [57]. Site-specific phosphorylation of tau could lead to tau mislocalization and accumulation. Phosphorylated-T231-tau mislocalizes to the somatodendrites and accumulates there, which is a prerequisite for the formation of tau tangles in neurogenic fibers [58]. But the opposite is also reported. Biophysical studies have recently revealed that phosphorylated-Ser356-tau (pS356-tau) can affect the stability of β6 and β7 structures in protofibrils of PHFs and straight filament, blocking the interaction between tau and Aβ peptides. Thus, pS356-tau alters the structure of PHFs and disrupts the formation of NFTs [59].

Intracellularly, NFTs formed by hyperphosphorylated tau proteins can lead to neurodegeneration and cell death, by activating calpain and causing mitochondrial dysfunction [60]. However, there are arguments that tau protects against cell death. For instance, phosphorylated tau antagonizes apoptosis by stabilizing β-catenin [61, 62]. Hyperphosphorylation of tau could also negatively regulate the physical function of nuclear tau when protecting DNA from damage [63]. This may suggest that phosphorylation at distinct tau residues and different contexts may cause diverse effects on downstream signals, which may alter the course of cell death [64].

### Acetylation

Acetylation belongs to one of the crucial PTMs of tau with various biological functions such as metabolism, histone regulation, and stress response. In transgenic AD mice models and AD patients, tau acetylation level is significantly increased [65]. Multiple research projects have focused on the potential pathogenesis and impact of tau hyperacetylation in AD.

Recently, tau acetylation at the lysine sites has been found significantly increased in brains with human tauopathies [66, 67] and influences numerous biological functions of tau, such as synaptic connection, mitochondrial function, tau aggregation, and diffusion of pathogenic tau [65, 67, 68]. Mitochondrial dysfunction is a clear early feature of AD [69]. Increased acetylation of tau at K274 and K281 has been identified in the brains of AD patients and has recently been found to have damaging effects on mitochondria [70]. However, BGP-15, a poly (ADP-ribose) polymerase (PARP) inhibitor and insulin sensitizer [71], is a potential therapeutic agent for AD as it mitigates these impairments in memory and learning via attenuating the aforementioned impairments caused by acetylation of tau at K274 and K281 [70, 72]. On the other hand, recent studies have shown that acetylation of tau sites K311 and K340 both reduced the rate of microtubule polymerization, so it may affect AD by promoting NFT formation [73]. Li et al. identified lysine residue acetylation patterns on tau fragments flanking the amyloidogenic motifs that contributed to fibril assembly and observed amyloid fibril structures composed of acetylated tau fragments. It suggests that specific lysine residue acetylation patterns cause pro-aggregation interactions of tau, which can assemble tau into different amyloid folds [74].

Tauopathies result in synaptic loss and are one of the earliest structure-related factors in cognitive dysfunction and AD progression. It is worth noting that the increase of phosphorylation and acetylation induces tau to mislocate the synapse, inhibit the release of synaptic vesicles, and affect the activity-dependent secretion of tau from neurons [75]. Therefore, the accumulation of acetylated tau at synapses has a negative effect on crucial neurons related to neuronal activity and synaptic function. Furthermore, most tau proteins are degraded through chaperone-mediated autophagy, while when tau undergoes acetylation, it is preferentially degraded through endosomal microautophagy and macroautophagy, in part because acetylated tau inhibits chaperone-mediated autophagy and leads to the extracellular release of tau [76].

cAMP-response element binding protein (CBP) mediates tau acetylation, while histone deacetylase 6 (HDAC6) and sirtuin 1 (SIRT1) mediate tau deacetylation [65]. According to reports, tau acetylation at the sites regulated by HDAC6 competes with tau phosphorylation, thus disrupting tau aggregation [50]. It has been found that HDAC6 inhibitor CKD-504 significantly alters the tau interactome in the brain of animal models with AD and brain organoids derived from AD patients [77]. Acetylated tau collects chaperone proteins including Hsp40, Hsp70, and Hsp110 to form a complex and then combines with new tau E3 ligases, including RNF14 and UBE2O [77]. Such tau interactome might degrade pathological tau via the proteasome pathway, thus improving cognitive capability and synaptic impairment of AD mice. Mounting evidence suggests that SIRT1 can protect neurons from neurodegenerative diseases including AD [78]. In the brains of patients with AD, the decrease of SIRT1 level elicits the hyperacetylation and accumulation of tau, thus aggravating the transmission of pathogenic tau [68, 79]. The latest research found that the activation of AMP-activated protein kinase (AMPK) reduces the tau acetylation level and attenuates memory damage, becoming a potential target for future AD treatment [80]. Furthermore, in tauP301S transgenic mice, a high level of SIRT1 significantly ameliorates the diffusion of tau pathology into anatomically associated brain regions, suggesting that SIRT1 not only regulates tau acetylation but also inhibits the propagation of tau pathology in vivo [81].

### Methylation

In common proteins, lysine residues can be methylated three times at most, resulting in a mono-, di-, or trimethylated lysine, which lead to different biological outcomes. Each time methylation is conducted, a proton would be removed from the ε-amino group, therefore reducing the hydrogen bond potential of the Lys. Thus, methylation could also increase the hydrophobicity and bulk of the Lys side chain [82].

Tau proteins can be mono- or dimethylated in both healthy or AD brains. Methylated tau may be involved in the pathogenesis of tauopathies, which is related to aging, tau aggregation, and changes in microtubule dynamics. One study found that tau aggregated in AD brains was monomethylated at seven Lys sites in the PRD and MBD, where the relative abundance varied between 12 and 67% [83]. Later, they discovered that methylated tau was widely distributed in the AD brain and co-localized with neurogenic fibers in late AD [83]. Several types of methylation mimetics with tau mutation P301L show impairment of microtubule binding and enhancement of prion-like seeding aggregation in frontotemporal dementia [84]. It is interesting that the methylation status of tau changes qualitatively with the progression of aging and disease [84]. There is a possibility that aging alone determines tau methylation, which is similar to the “aging clock” described by DNA methylation [85]. However, the result of this conversion may be pathological. Lysine methylation is a physiological PTM of tau protein which shifts from predominantly dimethyl lysine to monomethyl lysine along with the aging and progress of disease, and pharmacologically enhancing tau methylation may provide a way of preventing pathological tau aggregation [86]. In addition, several studies show that residue-specific methylated tau proteins are more likely to assemble into insoluble structures. methylation of K317 reduces tau solubility, which facilitates efficient dimerization of tau proteins and thus promotes more advanced oligomerization [87–89]. Besides, K317 methylation may also increase microtubule dynamics and somewhat inhibit tau-dependent proliferation of Hela cells [87, 90–92].

### Other PTMs

Other PTMs of tau include glycosylation, ubiquitination, and SUMOylation.

N-terminal glycosylation occurs in hyperphosphorylated tau proteins. The role of N-terminal glycosylation in AD is unclear, and some questions remain about its correlation with tau function [82]. Increased O-GlcNAcylization on serine and threonine residues on tau proteins may protect tau from being phosphorylated [93]; O-GlcNAcylization increases tau-microtubule interactions, increases tau degradation, and inhibits tau aggregation [94–96]. Therefore, O-GlcNAcylization of tau may have neuroprotective effects.

As a lysine-rich residue protein, tau is highly sensitive to ubiquitination. There are 17 residues of the 44 lysine residues from the 2N4R tau isoform that have been found to be ubiquitinated. And most of them are located in the MBD [97, 98]. Lysine 63 ubiquitinated tau oligomers play an important role in tau pathological aggregation, accumulation, and proliferation [99]. Insoluble tau from AD brains is predominantly ubiquitinated through K48-linked modifications [97], when soluble tau could also be ubiquitinated through K63 polyubiquitin coupling [100], which suggests that dissolvable and aggregated tau are degraded by different pathways [101]. There is no direct evidence to prove that tau ubiquitination has something to do with neurotoxicity. However, as AD progresses, proteasomal damage could lead to the accumulation of ubiquitinated proteins. It has been found that proteasome inhibition increases the accumulation and insolubility of tau proteins independent of tau phosphorylation and that proteasome inhibition may also indirectly lead to reduced relative tau phosphorylation in the rat brain through c-JUN terminal kinase (JNK) inhibition [102]. When E3 ligase attaches ubiquitin, deubiquitinases (dUbs) remove them. Cysteine protease Otub1 is the only dUbs reported to target tau, removing the K48 polyubiquitin chain from endogenous tau and preventing tau from degrading in transgenic mouse-derived primary neurons [103]. Overexpression of USP10, one of the important dUbs, directly leads to elevated levels of total and phosphorylated tau, inducing tau aggregation and delaying tau degradation [104].

SUMOylation may be a PTM that is highly correlated with tauopathies. SUMO1 modification, combining with tau truncation, may contribute to the pathogenesis of PSP [105]. Tau-SUMOylation could induce tau to be hyperphosphorylated at a series of AD-associated sites, and tau hyperphosphorylation can in turn promote its SUMOylation and inhibit tau degradation by reducing tau protein solubility and ubiquitination [106]. Phosphorylation of tau at residue S-214 promoted its SUMOylation at specific sites and improved its stability [107]. In an in vivo model of AD, few phospho-tau particles in neuritis plaques were ubiquitin-positive in cortical sections from Tg2576 mice, whereas all phospho-tau particles and punctate deposits were SUMO-1-positive [108].

## Role of Tau Fragments in Neurodegeneration

Truncation is one of the most important modifications in PTMs. Cleavage of tau by diverse proteolytic enzymes produces short, easily aggregated fragments that participate in the pathogenesis of neurodegenerative diseases such as AD, CBD, and PSP. Tau accumulation in neurons and glial cells is a crucial characteristic of tauopathies [6, 109]. Tau degradation is found to be the precipitating factor of tau accumulation, and different tau fragments are detected in CSF and brain extract [110, 111]. Tau in CSF is composed of a number of fragments, with the central region and N-terminal tau being the most affluent [112]. However, variants of CSF tau are of a complex variety, containing tau peptides from the N-terminal to the C-terminal, and the most reproducible phenomenon is a significantly increased level of C-terminal truncated tau in patients with AD [111, 113, 114]. Most CSF tau lack C-terminal and MBD parts, and the neuronal secretion of these tau fragments is induced by Aβ exposure, suggesting that CSF tau levels exhibit neuronal responses to Aβ pathology [115]. Numerous tau fragments cleaved by diverse proteolytic enzymes play different roles in tau pathology (Table 1).

**Table 1:** Main tau fragments involved in tau pathology

Type of fragments	Associated proteases	Found in CSF	Lesion	References
TauΔD421	Caspase-3, -6, -7	Reported	Axonal loss, mitochondrial dysfunction, tau polymerization, and aggregation in vitro	[116–119]
ΔTau314	Caspase-2	Not yet reported	Mislocalization to dendritic spine, reduction of AMPA receptor, and excitatory neurotransmission	[120, 121]
17 kDa tau	Calpain-1 and -2	Not yet reported	Altering the composition of cytoskeleton, synaptic degeneration through clathrin-mediated pathway, blocking axonal transport	[122, 123]
N224 tau	Calpain-2	Reported	Not clear (but worthy of diagnosis)	[124–126]
Tau1–368	AEP	Reported	Promoting aggregation, promoting the expression of Aβ, phosphorylation inducing memory impairment	[127–130]
Tau168–368	AEP	Not yet reported	Accumulation in microglial cells	[131]
26–230tau (NH2-tau), 26–44 NH2-tau	Caspase and calpain-1 and caplain-2	Reported	Damage of mitochondrial function, presynaptic defect, damage of study/memory function	[110, 132–137]
tau151–391, tau297–391	Unknown	Not yet reported	Form PHFs in vitro and cultured cells	[138, 139]

### Caspase

Caspases are a cysteine protease family that are primarily involved in cell death and inflammatory responses. On the one hand, caspase-8, -9, and -10 could initiate apoptosis by activating the executioner caspase-3, -6 and -7. On the other hand, caspase-1, -4, -5 and -11 are regarded as inflammatory caspases [140]. Especially, activated caspase-3 could lead to non-inflammatory cell death [141–143]. Caspase-3-cleaved tau could be possible markers of preclinical neurodegenerative disease [144]. The substrate for caspase-6 is lamin A, and its cleavage is crucial for apoptotic chromatin condensation [145]. Degenerative diseases such as AD are also associated with neuronal apoptosis and inflammation [146, 147], so it can be assumed that there is a relationship between caspases and the progression of AD. Activation of caspase induces the formation of NFTs via cleaving tau. Truncated tau cleaved by activated caspases recruits normal tau to form tangles, which in turn reduce caspase activity to inhibit acute neuronal death and make tangle-bearing neurons long-lived [148, 149]. Caspase-cleaved human tau fragments (tauΔD402, and tauΔD421) are proved in the pathogenesis of tau and induce cognitive impairment [16, 150].

#### TauΔD421

TauΔD421 is produced by caspase-3, caspase-6, and caspase-7, whereas tauDΔ402 is produced only by caspase-6. Tau is cleaved at aspartate 421 (D421) by these caspases to form tauΔD421. In individuals without cognitive impairment, active caspase-6 is present only in the regions where NFTs first appear: hippocampal CA1 and the entorhinal cortex [151]. Tau fragments cleaved by caspase-6 at amino acids 421 (tauΔD421), as well as active caspase-6, are present in pre- and mature NFTs composed mainly of tau filaments in the patients’ brains with mild cognitive impairment (MCI) or early stage of AD, while absent in the non-AD brains [150, 152]. Levels of tauΔD421-positive CA1 are negatively associated with mini-Mental State Examination scores, and the levels of serum distinguish patients with AD and MCI from those with other dements [116, 153].

Numerous studies have demonstrated that tauΔD421 participates in the pathological mechanisms of tau in many aspects, inducing axonal loss, mitochondrial dysfunction, and tau polymerization and aggregation in vitro [116–119]. TauΔD421-induced alterations in mitochondrial dynamics could abruptly affect normal synaptic communication. In AD mice, cells expressing tauΔD421 show fragmented mitochondria and impaired mitochondrial dynamics by inhibiting the optic atrophy protein (Opa1) [154]. In addition, tauΔD421 inhibits mitochondrial transport by increasing the co-localization of trafficking kinesin-binding protein 2 (TRAK2) with mitochondria and reducing ATP production, which in turn reduces the number of transporting mitochondria, leading to the deposition of mitochondria in the cytoplasm and a reduction of synaptic mitochondria, which leads to synaptic failure in AD [155].

Immunodepleting of tauΔD421 inhibits the aggregation of tau induced by high molecular weight protein fraction in AD brain, indicating that tauΔD421 affects the pathological spread of tau [156]. Furthermore, tauΔD421 may lead to tau oligomers, microgliosis, and neurodegeneration [148, 157]. However, if a tau mutant that cannot be cleaved by caspase in D421 is expressed, mice could develop memory deficits, synaptic plasticity defects, and prepathological tau changes [158]. The accumulation of tauΔD421 correlates with increasing age. Overexpression of tauΔD421 in middle-aged mice contributes to significant hippocampal long-term potentiation deficits and cognitive deficits, and the increasing age is positively associated with the neuronal degeneration affected by tauΔD421 accumulation [159]. Moreover, the apoptosis of rat cortical neurons is initiated, when treated with aggregated Aβ, and during the process, tau is cleaved at D421 with the onset of neurite death, indicating that tau cleavage is an important component of the deleterious cascade resulting in neuronal dysfunction and apoptosis [160]. Fasulo et al. found that tau151–421 cleaved by caspase-3 exerted significant apoptotic effects on rat hippocampal neurons [161]. But how the D421 could still remain in undead neurons and how it reverses the apoptotic effect and found in the AD brain are still unknown. TauΔD421 impairs neuronal firing, causing inefficient initiation of network bursts, consistent with reduced excitatory drive. Since reduced neuronal activity is coupled to proteasome dysfunction, it drives cleaved tau accumulation at the post synaptic density and subsequent synaptotoxicity [162].

As to possible treatment for this target, a study reveals that tauΔD421 expression significantly reduces dendritic spine density and synaptic vesicle number in hippocampal neurons. Also, neurons transfected with tauΔD421 showed a significant accumulation of synaptophysin protein in soma. All these synaptic changes could be prevented by cyclosporine A, a drug which could inhibit the function of mitochondrial permeability transition pore (mPTP). It means that mPTP may play a role in synaptic dysfunction derived from caspase-3-cleaved tauΔD421, and cyclosporine A may have a cure for AD and other neural dysfunctional diseases [163].

#### ΔTau314

Aspartate 314 (D314) of tau is another site for cleavage by caspase-2; this process could mislocalize tau to dendritic spines, which can facilitate the mislocalization of tau proteins, leading to reduced postsynaptic AMPA receptors and reduced excitatory neurotransmission [120], thereby affecting cognitive and synaptic function in animal and cellular models of tauopathies and reversibly damaging memory in animal models [121]. The soluble fragment Δtau314 brought about by this cleavage is significantly elevated in the brains of individuals with Huntington’s disease, Lewy body disease, and AD [120, 164], so Δtau314 could become a CSF biomarker to predict tauopathies. Δtau314 protein has now been found to have a distribution in the human striatum and prefrontal cortex [164] and has also been found to be present in the inferior temporal gyrus [165]. Using caspase-2 inhibitors could block the production of Δtau314, inhibiting the excessive dendritic spinal accumulation of tau and thus influencing excitatory neurotransmission in reverse in cultured primary hippocampal neurons of rats [166]. Another treatment for this target is the D314E mutation. Targeted insertion of D314E mutated htau into mice to express cleavage-resistant D314E mutant retards transgene-mediated accumulation of tau in postsynaptic densities [167].

### Calpain

Calpains are a series of calcium-dependent proteases that are widely expressed in a variety of organisms and exert activity under neutral pH conditions [168]. Calpain activation is a crucial neurodegenerative factor leading to apoptosis and is closely associated with pathological processes of neurodegenerative diseases [169]. There is always a link between neurodegenerative diseases like AD, Huntington’s disease and PD, and an abnormal increase in calcium concentration, which leads to the abnormal activation of calpain [170]. Abnormal activation of calpain can nonspecifically cleave a variety of target proteins including tau proteolysis. Specifically, calpain-1 and -2 lead to the cleavage of 17 kDa tau, and calpain-2 mediates the formation of N224 tau [171, 172], both of which are common tau fragments with neurotoxic effects and mediation in the progression of degenerative diseases. Focus on possible treatment, calpain-1 activity may be downregulated by UB-ALT-EV, a new NMDA receptor antagonist, under which 5FXAD mice show a better cognitive performance, revealing that UB-ALT-EV could reduce cognitive alterations in the mice model of familial AD [173].

#### 17 kDa tau (Tau45–230)

β-Amyloid oligomers could induce calpain activation and thus cleavage of tau. One of the more studied fragments is the 17 kDa tau fragment comprising 45–230, which arises due to the cleavage by calpain-1 at K44–K45 [174] and cleavage by calpain-1 and -2 at R230–T231 [172]. Tau45–230 occurs in the temporal cortex of AD patients and can also be detected in the brains of patients with other tauopathies such as Pick’s disease and Parkinson’s syndrome-17 [175]. Tau45–230 may exert toxicity by way of oligomerization. The oligomer can be incorporated by neurons and indirectly induce neurosynaptic degeneration. In vitro, tau45–230 can oligomerize to form heptamers and octamers [176]. Another study reported that tau45–230 oligomers can be internalized by cultured hippocampal neurons and induce neurosynaptic degeneration through a clathrin-mediated mechanism [177].

Tau45–230 can also function as a toxic agent by altering the composition of the normal neuronal cytoskeleton and affecting neurite growth and transport. In cultured hippocampal neurons, tau45–230 can exert toxicity by partially blocking axonal transport along the microtubules, markedly reducing the organelles transported along axons [178]. Tau45–230 can also trigger a transient increase in unstable tyrosine microtubules [123]. Significant synaptic deficits were detected in mice expressing tau45–230 at 6 months postnatally, accompanied by altered NMDA receptor expression [122]. Tau45–230 overexpression leads to apoptosis in Chinese hamster ovary cells CHO, hippocampal neurons, and mouse hippocampus [122, 179, 180]. Yet it has also been argued that the 17 kDa tau fragment may be actually a shorter tau (125–230) fragment [172]. The accumulation of both tau125–230 and tau45–230 fragments has recently been found in brain tissue from acute ischemic shock [181]. Thus, the 125–230 fragment may also be involved in pathological progression of neurodegenerative disease.

#### N224 tau

Tau in CSF is recently found to have a major calpain-2-induced cleavage at amino acid (aa) 224, and the N-terminal tau fragment terminating at aa224 (N224) is prominently upregulated in AD [182]. Levels of N224 tau from the soluble brain fraction of AD patients are significantly lower than that of controls, while in CSF, N224 tau is higher in AD patients than in controls [124]. High levels of N224 tau correlate with low Mini-Mental State Examination (MMSE) scores [126]. Cicognola et al. demonstrated that increased levels of N224 tau in CSF were connected with conversion from MCI to cognitive decline and AD [124]. Moreover, N224 tau levels are lower in PSP or corticobasal syndrome (CBS) than in AD [125]. N224 tau exists in tangles and neuronally derived extracellular vesicles (NDEV), with neuron-specific secretion in CSF and upregulation in AD, indicating its involvement in cognitive impairment and AD pathology [124]. In addition, a recent study has revealed that levels of N224 tau in AD patients were higher than that in MCI, subjective cognitive decline SCD, PD, PD dementia, multiple system atrophy, and PSP [126]. Therefore, N224 tau could contribute to distinguish AD from SCD and other dementias and may be an important tau biomarker to study species of tau fragments in CSF.

#### NH2-tau

NH2-tau is a 20–22 kDa fragment that predominantly involves in mitochondria from synaptosomes of the AD brain [134]. This fragment was discovered by Corsetti et al. while studying the toxic effects of the N-terminal fragment of tau. At that time, they found that some NH2-terminal fragments, such as 26–44 tau and 26–230 tau, induced *N*-methyl-D-aspartate receptor (NMDAR)-mediated cell death [183], and 26–230 tau was also found by others in cellular and animal models of AD, so they named this fragment NH2-26–230 tau (aka NH2-tau) [184]. The full length of NH2-tau comprises 26–230 aa, and the first 25 amino acids have been clearly sheared down by caspases, while amino acids after 231 are sheared by calpain-1 and -2 [185, 186]. NH2-tau is an essential component in the toxic cascade that leads to neurodegeneration or cell death [186]. Following apoptotic stimulation or neurodegenerative injury, aberrant activation of cysteinyl asparaginase may produce toxic NH2-tau fragments which could then propagate and also elaborate cellular dysfunction.

NH2-tau enrichment in hippocampal parenchyma revealed significant impairment of learning/memory capacity in treated healthy mice, which was associated with reduced synaptic linkages and neuroinflammatory responses [132, 133]. NH2-tau can accumulate in AD synapses and be detected in CSF [110], and this truncated tau protein in vitro can acutely cause presynaptic defects in K^+^-induced glutamate release from hippocampal synaptosomes as well as altered local calcium dynamics [133]. Downregulation of the CREB/c-fos pathway can be detected in the hippocampus following subchronic intracerebroventricular infusion of NH2-tau protein into wild-type mice [132]. A more widely reported function of NH2-tau is its damage to mitochondria. Extracts of Aβ oligomers may damage mitochondrial function by producing this kind of fragment in vitro in rat hippocampal neurons and mature human SY5Y cells [134]. NH2-tau can impair mitochondrial autophagy, directly through the inhibition of ADP/ATP exchange depending on ANT-1 [135, 137, 187] and indirectly through impairing mitochondrial autophagy [135]. One of the pathways is that NH2-tau causes abnormal recruiting of parkin and UCHL-1 to mitochondria, where deleterious autophagic clearance occurs [187].

NH2 26–44 tau acts as the minimum active moiety of NH2-tau. Experiments with the NH2-derived NH2 26–44 tau peptide revealed that both cytochrome C oxidase and adenine nucleotide translocators are targets of this fragment, but adenine nucleotide translocation is a characteristic target of the fragment, and this action significantly affects cellular access to ATP synthesized by mitochondria [136, 137]. In contrast, fragments 1–25 do not have such a significant toxic effect. Another example is that this truncated tau protein accumulates at synapses of AD individual and can be secreted into parenchyma. This could acutely trigger presynaptic defects in glutamate release evoked by K^+^ at synaptosomes in the hippocampus as well as alter local Ca^2+^ dynamics [133].

It has recently been shown that 12A12mAb can well neutralize NH2tau fragments [188], restoring synaptic linkage and cytoskeletal function and effectively improving learning and memory function in pathological AD mice [189]. NH2-tau proteins may be involved in pathological degeneration of the retina, but targeted injection of monoclonal antibodies can ameliorate the degenerative process [188] and improve visuo-spatial recognition memory [190]. Interestingly, 12A12mAb could downregulate the steady state expression levels of APP and beta-secretase 1 (BACE-1), thus limiting the Aβ production both in the hippocampus and retina, showing that 12A12mAb could also make an influence on APP involved pathological mechanisms [191].

### AEP

Asparagine endopeptidase (AEP) is a lysosomal cysteine proteinase, also named δ-secretase, which is not expressed in neurons but in microglia [131, 192]. AEP specifically hydrolyses the carboxyl-terminal peptide bond of asparagine residues and is a crucial enzyme participating in the processing of antigens and autoantigen processing, since it is known to involve in the processing of antigens for MHC class II presentation in the lysosomes of antigen-presenting cells [193, 194]. A decrease in pH can activate inactive full-length pro-AEP [194]. AEP is related to neurodegeneration [192, 195]; it could mediate dementia by enhancing amyloid plaque and tau hyperphosphorylation, indicating that it played an important role in neurodegeneration [196]. It can specifically shear human synuclein (aSyn) at the N103 site and trigger its aggregation to produce neurotoxicity and mediate PD pathogenesis [197]. In the brain of patients with PD, Netrin-1 expression is downregulated, and UNC5C receptors are specifically sheared by activated AEP, leading to apoptosis of dopaminergic neurons [198]. In AD, AEP activity is upregulated, and the activation of AEP leads to hyperphosphorylation of tau protein, mediating the neurofibrillary pathology [192, 197, 198]. Tau168–368 and tau1–368 are important fragments processed by AEP.

#### Tau168–368

Tau is cleaved at N167 and N368 by AEP after endocytic uptake into microglia rather than neurons, forming tau168–368 fragment at similar concentrations in both AD and control groups, which does not accumulate in microglia [131]. AEP-mediated tau cleavage does not alter in AD compared with control groups. Therefore, AEP-cleaved tau168–368 is a component of the proteolytic cascade that induces tau degradation, and AEP-mediated cleavage of tau is a physiological response that occurs when secreted neuronal protein is degraded in microglia. Additionally, the amount of tau fragment cleaved at the site N368 in insoluble tau aggregates is very small (< 0.1%) compared with uncleaved tau, indicating that AEP-cleaved tau has a limited role in tau aggregation in AD [199]. Based on these results, AEP-mediated tau cleavage might not be a direct valid therapeutic target for AD. However, AEP-cleaved tau does participate in other important aspects of AD pathology.

#### Tau1–368

To date, effective fluid biomarkers for tau pathology in AD are still lacking. A new SIMOA® quantification assay has recently been established to assess the presence of tau1–368 in CSF. The results showed that tau1–368 is a tangle-rich fragment, and the tau1–368/total tau (*t*-tau) ratio is significantly decreased in AD patients, reflecting tangle pathology. This ratio in CSF is negatively associated with the retention of 18F-Genentech Tau Probe 1 (GTP1) [200]. This new tau biomarker might aid in the AD diagnosis and contribute to drug development targeting tau pathology. In addition to cleaving tau at N368, AEP can also cleave amyloid precursor protein (APP) to form APP 586–695, contributing to cognitive impairment and the pathogenesis of AD [127, 128]. Upon APP stimulation, tau1–368 significantly enhances beta-secretase1 (BACE1) expression and Aβ production, facilitating its nuclear translocation [127]. Notably, the activation of JAK2 or SGK1 kinases by Aβ phosphorylates STAT1 and triggers its binding to tau1–368 [201]. Thus, not only tau may be a downstream effector of Aβ, but also tau1–368 can exacerbate Aβ production. Notably, aberrant PTMs of truncated tau are equally important in endogenous tau pathology and neurodegeneration. Tau1–368 contains MBD and PRD, whose phosphorylation stimulates endogenous tau phosphorylation and aggregation. Interestingly, phosphorylated tau1–368 causes body weight loss and recognition memory impairment of C57BL/6 J mice, whereas non-phosphorylated tau1–368 does not [129, 130]. In addition, overexpression of phosphorylated tau1–368 elicits hippocampal neuronal loss and gliosis, but tau1–368 delivery and tau1–368-mediated neurotoxicity are phosphorylation independent [129, 192]. Spinal cord injury could stimulate AEP activation in mice. Activated AEP then cleaved APP and tau, resulting in tau1–368 formations, and consequentially accelerated Aβ deposit and Tau hyperphosphorylation, resulting in cognitive impairment [196].

## Secretion and Transmission of Truncated Tau

The aggregation and diffusion of misfolded tau protein in the CNS is one of the hallmark features of tauopathies. In AD brains, tau aggregates accumulate first in the trans-entorhinal cortex and then spread to the anatomically connected hippocampus, leading to progressive cognitive deficits and neurodegeneration [202]. Numerous studies have revealed that tau can undergo prion-like pathological propagation between cells [203], that is, cellular secretion of tau protein, internalization of tau, and misfolding of normal tau in recipient cells, but currently, no epidemiological evidence shows that tau aggregates are infectious [204, 205]. Specifically, prion-like transmission of tau starts with the production of transmissible seed-competent tau monomers [206], which generate distinct biologically active and self-replicating assemblies called strains in distinct tauopathies. Secondly, pathological cells secrete and normal cells subsequently uptake seed-competent tau. Finally, templated tau misfolding occurs in recipient cells, resulting in the formation of new tau seeds. Under physiological and pathological conditions, tau seeds are actively secreted outside the cell through a variety of possible unconventional pathways, such as plasma membrane translocation, secretion based on membrane organelles, and ectosomal shedding [207–209]. The physiological role of extracellular tau needs to be further investigated, but preliminary studies revealed that tau in the extracellular space enhances electrical activity in primary neuronal cultures [210].

Hyperphosphorylation can promote tau detachment from microtubules and cell secretion of full-length tau [211]. Recently, however, truncated tau has been found to be transmitted between cells in a limited capacity, independent of phosphorylation. Although phosphorylation does not affect the cell-to-cell transmission of tau1–368, it mediates neurotoxicity when tau1–368 is over-expressed in the hippocampal CA1 region, resulting in anxiety, impaired recognition memory, and weight loss [129]. In addition, tau fibrils synthesized artificially with truncated tau were sufficient to deliver tau inclusions in mouse models. To be specific, synthetic preformed fibrils (pffs) assembled from truncated tau containing four microtubule-binding repeats induce the time-dependent propagation of NFT-like inclusions from the injection site to contiguous brain regions [212].

Tau degradation is normally completed via the ubiquitin–proteasome system and autophagy-lysosome system [213, 214]. However, phosphorylated tau in pathological states can be directly degraded through the autophagy-lysosome system [215]. As a further step, excess tau remains on the surface of the lysosomal membrane, which makes it possible for tau to oligomerize at or near the organelle surface [102, 216]. The accumulation of aggregated proteins could result in lysosomal dysfunction in multiple ways, including membrane rip and lysosomal cleavage, which can promote tau aggregation to form a vicious cycle. It is probable that lysosomal dysfunction results in the compensatory release of extracellular vesicles/exosomes in neurodegenerative diseases [217, 218]. As tau lacks conventional signal sequences, there are four possible unconventional protein secretion mechanisms leading to tau secretion [219]: (1) direct export across the plasma membrane; (2) secretion via ectosomes that are shed from the plasma membrane, or microvesicle shedding; (3) formation of intraluminal vesicles (ILVs) through late endosome membrane’s inward budding, free tau in cytosol can be sequestered in ILVs which could form endosomes of multivesicular vesicles (MVBs), and subsequently, tau can be packaged into exosomes and secreted extracellularly upon fusion of the MVB with the plasma membrane; and (4) direct release into the extracellular space in a vesicle-free manner via the organelle hitchhiking pathway.

To be inclusive, tau can enter extracellularly via ectosome, exosome, or without vesicles [220]. Tau-containing exosomes are derived from MVB and in AD patients, where exosomes have significantly higher levels of tau protein than normal exosomes [221]. Exosomal tau from neurons is hyperphosphorylated, and the release of tau-containing exosomes could be promoted through depolarization of neurons. These exosomes complete the transmission of tau between neurons through synaptic connection [222]. In human-induced multifunctional neurons derived from stem cell, the exosomes contain mutant tau (mTau) and contain the P301L and V337M mutations. In Podvin’s study, mTau is a dynamic regulator of exosome biosynthesis, leading to the acquisition, deletion, upregulation, or downregulation of protein cargo, resulting in pathological mTau exosomes that can cause p-tau neuropathology in the brain of mice intracellularly [223]. Microglia could also release tau-contained exosomes and cause the spread of abnormal tau. This kind of immune cell engulfs neurons containing pathological tau proteins, which are subsequently transmitted to exosomes involved in the pathological transmission of tau in the brain. This transmission can lead to tau propagation from the olfactory cortex to the dentate gyrus region. However, clearing microglia and inhibiting exosome synthesis with GW4869 is able to limit tau propagation in patient brains [224]. P2RX7 is an ATP-gated cation channel that triggers exosome secretion, and oral administration of a P2RX7 inhibitor to P301S tau transgenic mice inhibited exosome secretion; thus, MC1 + and Alz50 + tau proteins were significantly reduced in the hippocampus, and significant improvements in working and situational memory of mice were discovered [225]. Truncated tau species which lack MBD that is essential for seeding have been reported to undergo active secretion.

Both tau fragments or full-length tau could be secreted. The PRD of tau could contribute as a membrane-binding site when full-length tau was secreted [226]. However, extracellular species are predominantly composed of N-terminal fragments of tau, while there is no evidence that C-terminal tau fragments are involved [210]. The effect of expressing constructed N-terminal and full-length tau in transfected neuronal lines is examined, and secreted tau shows a cleavage pattern which is similar to tau in CSF from AD patients [227]. Htau was secreted by neuronal and non-neuronal cells when htau was upregulated. In HeLa cells, phosphorylation and cleavage of tau favored its secretion. A mutant form of tauΔD421, where caspase-3 prefer to cleave, was secreted more than wild-type tau, which could contribute to the pathological tau propagation in brain and its accumulation in the CSF [228].

The transcription factor EB (TFEB), a master of regulating lysosomal biogenesis, regulates the secretion of truncated mutant tau lacking MBD. Since lysosomal exocytosis mediated by TFEB could promote cellular clearance, it has been proved that reduced interstitial fluid tau without TFEB’s occurrence have something to do with enhanced cell-to-cell pathology and accelerated spreading; thus, exocytosis could act as a clearance mechanism, reducing intracellular tau on pathological conditions [229]. VAMP8, a late endosomal R-SNARE, increased tau secretion, which was cleaved at the C-terminal. Upon VAMP8 overexpression, an increase of caspase-3-cleaved tau in the cell lysate and medium was observed. However, this intracellular cleaving may not be necessary, since extracellular tau cleavage by caspase-3 could occur resulting from released active caspase-3, which reached the most when VAMP8 was upregulated [230]. This could explain why full-length tau could be secreted, but most of extracellular tau are C-terminal cleaved. Tau cleavage mediated by AEP is a physiological event occurring during secreted neuronal proteins are degraded in microglia [131]. N-224 tau fragment is specifically processed in neurons, discovered in NFT, secreted in CSF, and overexpressed in AD [231].

Few articles have discussed tau-containing ectosomes, but in principle, similar biogenesis mechanisms can be activated in distinctive regions of the cell to produce ectosomes and exosomes. For example, the endosomal sorting complex required for transport (ESCRT) mechanism participated in the biosynthesis of both exosomes and ectosomes, and the situation is similar for both exosomal and endosomal sorting mechanisms [232].

## Conclusion

Tau protein is widely distributed within the neurons and has multiple functions, from modulating the cellular structure to regulating the expression of DNA or RNA, from connecting the transported organisms and the microtubules to controlling the transportation between nucleus and plasma. Tau is one of the most crucial molecules in tauopathies like AD, PSP, and CBD. Numerous studies proved tau structural abnormalities were often induced by post-translational modifications and abnormal protease cleavage, where they contribute to induce pathological lesions in the nervous system. The special role and pathological mechanism of tau abnormalities in these diseases are very complex and unclear till now. In fact, the precise understanding of whether tau is the causative factor underlying these diseases or a consequence of other pathogenic agents also remains elusive. Nevertheless, more in-depth investigation is still worthy of attention and expectation because that tau and its fragments can serve as fundamental basis for tauopathies diagnosis, novel biomarkers for assessing disease progression, and a potential target for therapeutic interventions.

## Data Availability

Not applicable.
